# Microarray studies on effects of *Pneumocystis carinii *infection on global gene expression in alveolar macrophages

**DOI:** 10.1186/1471-2180-10-103

**Published:** 2010-04-08

**Authors:** Bi-Hua Cheng, Yunlong Liu, Xiaoling Xuei, Chung-Ping Liao, Debao Lu, Mark E Lasbury, Pamela J Durant, Chao-Hung Lee

**Affiliations:** 1 Department of Obstetrics and Gynecology, Chang Gung Memorial Hospital - Kaohsiung Medical Center, Chang Gung University College of Medicine, Kaohsiung, Taiwan; 2 Graduate Institute of Clinical Medical Sciences, Chang Gung University College of Medicine, Kaohsiung, Taiwan; 3 Division of Biostatistics, Department of Medicine, Indiana University School of Medicine, Indianapolis, IN 46202, USA; 4 Center for Computational Biology and Bioinformatics, Indiana University School of Medicine, Indianapolis, IN 46202, USA; 5 Center for Medical Genomics, Indiana University School of Medicine, Indianapolis, IN 46202, USA; 6 Department of Biochemistry and Molecular Biology, Indiana University School of Medicine, Indianapolis, IN 46202, USA; 7 Department of Pathology and Laboratory Medicine, Indiana University School of Medicine, Indianapolis, IN 46202, USA; 8 Department of Surgery, Teda Hospital, Tianjin, China; 9 Graduate Institute of Clinical Medical Science and Department of Laboratory Medicine, China Medical University, Taichung, Taiwan

## Abstract

**Background:**

*Pneumocystis *pneumonia is a common opportunistic disease in AIDS patients. The alveolar macrophage is an important effector cell in the clearance of *Pneumocystis *organisms by phagocytosis. However, both the number and phagocytic activity of alveolar macrophages are decreased in *Pneumocystis *infected hosts. To understand how *Pneumocystis *inactivates alveolar macrophages, Affymetrix GeneChip^® ^RG-U34A DNA microarrays were used to study the difference in global gene expression in alveolar macrophages from uninfected and *Pneumocystis carinii*-infected Sprague-Dawley rats.

**Results:**

Analyses of genes that were affected by *Pneumocystis *infection showed that many functions in the cells were affected. Antigen presentation, cell-mediated immune response, humoral immune response, and inflammatory response were most severely affected, followed by cellular movement, immune cell trafficking, immunological disease, cell-to-cell signaling and interaction, cell death, organ injury and abnormality, cell signaling, infectious disease, small molecular biochemistry, antimicrobial response, and free radical scavenging. Since rats must be immunosuppressed in order to develop *Pneumocystis *infection, alveolar macrophages from four rats of the same sex and age that were treated with dexamethasone for the entire eight weeks of the study period were also examined. With a filter of false-discovery rate less than 0.1 and fold change greater than 1.5, 200 genes were found to be up-regulated, and 144 genes were down-regulated by dexamethasone treatment. During *Pneumocystis *pneumonia, 115 genes were found to be up- and 137 were down-regulated with the same filtering criteria. The top ten genes up-regulated by *Pneumocystis *infection were Cxcl10, Spp1, S100A9, Rsad2, S100A8, Nos2, RT1-Bb, Lcn2, RT1-Db1, and Srgn with fold changes ranging between 12.33 and 5.34; and the top ten down-regulated ones were Lgals1, Psat1, Tbc1d23, Gsta1, Car5b, Xrcc5, Pdlim1, Alcam, Cidea, and Pkib with fold changes ranging between -4.24 and -2.25.

**Conclusions:**

In order to survive in the host, *Pneumocystis *organisms change the expression profile of alveolar macrophages. Results of this study revealed that *Pneumocystis *infection affects many cellular functions leading to reduced number and activity of alveolar macrophages during *Pneumocystis *pneumonia.

## Background

*Pneumocystis *pneumonia (PCP) is the most common opportunistic disease in AIDS patients [1,2]. During the early stage of the AIDS epidemic, PCP occurred in 60-80% of HIV infected patients in the United States and Western Europe [3]. Characteristic pathology features of PCP include infiltration of inflammatory cells in the lung, thickened alveolar septa, and foamy exudates in the alveoli.

Since *Pneumocystis *has a typical morphology of protozoa, it was initially considered as protozoa. It is now classified as a fungus because the composition and structure of its cell wall [4,5] and nucleotide sequences are more similar to those of fungi than to those of protozoa [6-9]. Although *Pneumocystis *organisms are found in many different species of mammals, they are strictly species specific [10]. Therefore, *Pneumocystis *from different host species has different names [11]. Among the more common ones, human *Pneumocystis *is called *Pneumocystis jirovecii*. Rat *Pneumocystis *is referred to as *P. carinii*; another rat *Pneumocystis *strain is called *P. wakefieldii*. Mouse *Pneumocystis *is named *P. murina*.

In immunocompetent humans and animals, alveolar macrophages (AMs) protect the hosts against *Pneumocystis *infection by actively removing this extracellular organism from the alveoli. However, AMs from *Pneumocystis*-infected animals are defective in phagocytosis [12,13], and the number of AMs in humans and animals with PCP is reduced [14-16]. These two defects impair the innate immunity against *Pneumocystis *infection. The reduction in alveolar macrophage (AM) number is mainly due to increased rate of apoptosis [17]. A recent study demonstrates that increased levels of intracellular polyamines trigger this apoptosis [18].

The increase in polyamine levels in AMs is due to increased de novo synthesis and uptake of exogenous polyamines [19]. Very little is known about the defect in phagocytosis during PCP. Decreased expression of macrophage receptors such as mannose receptor is a possible cause [20]. In this study, we used DNA microarrays to study global gene expression in AMs from *P. carinii-*infected rats to better understand the mechanisms of pathogenesis of PCP.

## Methods

### Rat PCP Model

*P. carinii *infection in rats was established as described previously [21]. Briefly, female Sprague-Dawley rats (Harlan, Indianapolis, IN) of 120-140 g were divided into three groups designated Normal, Dex, and Dex-Pc rats. Normal rats were immunocompetent and uninfected. Since rats must be immunosuppressed in order to develop PCP upon inoculation of *Pneumocystis *organisms, they were immunosuppressed by giving dexamethasone (1.8 mg/liter) continuously in drinking water to reduce the number of CD4+ T lymphocytes. These rats were referred to as Dex rats. Although the Dex rats were continuously immunosuppressed for nine weeks, they showed no signs of disease. Dex-Pc rats were Dex rats transtracheally inoculated with 7.5 × 10^6 ^*P. carinii *organisms one week after initiation of immunosuppression. To prevent other opportunistic infections, immunosuppressed rats were given 10,000 units of penicillin (Butler, Dublin, OH) weekly by intramuscular (i.m.) injection. All *P. carinii*-infected rats showed signs of PCP including weight loss, dark eyes, hunched posture, and respiratory distress eight weeks after inoculation of the organisms and were sacrificed for isolation of AMs. Age-matched Normal rats were used as controls, while age-matched Dex rats were used to control for effect of the steroid treatment. Giemsa and silver staining of lung impression smears was performed to determine the existence of *Pneumocystis *and other microorganisms. Any lungs that contained other microorganisms were excluded. All animal studies were approved by the Indiana University Animal Care and Use Committee and supervised by veterinarians.

### Isolation of alveolar macrophages

Rats were anesthetized by i.m. injection of 0.1 ml ketamine mixture (80 mg/ml ketamine hydrochloride, 0.38 mg/ml atropine, and 1.76 mg/ml acepromazine) and then sacrificed. The thoracic cavity and trachea were exposed by dissection. Bronchoalveolar lavage fluid (BALF) was obtained by instilling 5 ml of sterile phosphate buffered saline (PBS) one at a time into rat lungs with a 14-gauge angiocath (BD Biosciences, Bedford, MA) and then recovered until a total of 50 ml BALF was obtained [22]. The cells in this 50-ml BALF were pelleted by centrifugation at 300 × *g *for 10 min and then resuspended in 5 ml of Dulbecco's Modified Eagle Medium (DMEM). AMs were isolated by adherence on plastic tissue culture dishes at 37°C with 5% CO_2 _for 1 hr followed by washing with warm PBS three times to remove unattached cells. The purity of AMs was greater than 97% as determined by anti-RMA staining described previously [23].

### Isolation of RNA from alveolar macrophages

AMs from four each of Normal, Dex, and Dex-Pc rats were used. Total RNA was isolated individually from each sample using the RNeasy kit (Qiagen) according to manufacturer's instructions. Approximately 2 × 10^6 ^cells from each animal were used. The cells were washed with PBS and then lysed with 350 μl of Buffer RLT in the kit. The cell lysate was passed five times through a 20 gauge needle and then through the Genomic DNA Eliminator spin column by centrifugation, followed by addition of 350 μl of 70% ethanol to the flow through. The mixture was transferred to an RNeasy spin column placed in a 2 ml collection tube. The flow-through was discarded after a 15 s centrifugation at 8000 × *g*. The column was washed with 700 μl of Buffer RW1 and then with 500 μl of Buffer RPE twice. Total RNA was eluted from the column with 30 μl of RNase-free water and quantified by spectrophotometer.

### Microarray analysis

The Affymetrix GeneChip^® ^RG-U34A, containing 8799 rat genes and EST sequences, was used for the microarray analysis. Briefly, 2.5 μg of total RNA from each rat was reversely transcribed, using the standard 3'IVT protocol as described previously [24], and hybridized to a GeneChip. A total of 12 GeneChips were used, four for each sample group from Normal, Dex, and Dex-Pc rats. The data were first analyzed with Microarray Suite version 5.0 (MAS 5.0) using Affymetrix default analysis settings and global scaling as normalization method. The trimmed mean target intensity of each array was arbitrarily set to 1000. Comparisons of global gene expression and identification of genes that were up- or down-regulated by dexamethasone treatment or by *P. carinii *infection in AMs from the three different groups of rats (Normal, Dex, and Dex-Pc) were performed with the Partek Genomic Suite 6.4 Software (Partek Inc., St. Louis, MO). Identification of cellular functions affected by dexamethasone or *Pneumocystis *infection was achieved by using the Ingenuity Pathway Analysis (IPA) software (Ingenuity Systems Inc. Redwood City, CA). The microarray data generated in this study have been deposited in the Gene Expression Omnibus with the accession number GSE20149.

### Real-time RT-PCR

Approximately 0.2 μg of each total AM RNA sample was reversely transcribed to cDNA using the iScript cDNA synthesis kit (Bio-Rad, Hercules, CA) and random primers in a total reaction volume of 20 μl. The reaction mixtures were incubated at 25°C for 5 min, 42°C for 30 min, and 85°C for 5 min. Of this, 2 μl of each cDNA product was used for quantitative PCR analysis. Real-time RT-PCRs for various target genes were performed using the Assays-on-Demand™ gene expression kits. Each kit contained two unlabeled PCR primers and a FAM™-labeled TaqMan probe (Applied Biosystems, Foster City, CA). Since the expression of the ribosomal protein S8 (RPS8) is not affected by *Pneumocystis *infection, RPS8 mRNAs were assayed in an identical manner as an internal control as described previously [25].

## Results

### Quality of microarray data

**Figure 1:**
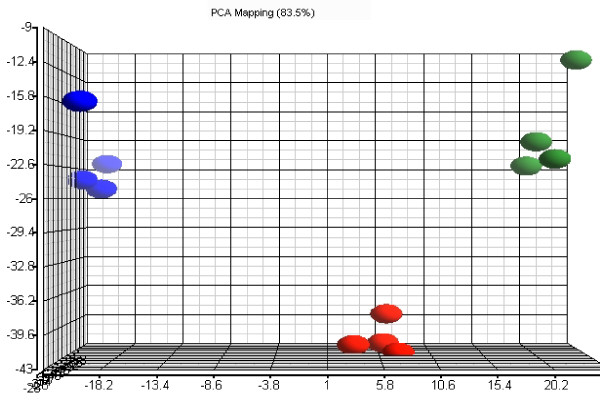
**Principle component analysis of microarray results**. The blue, red, and green oval dots represent linear combinations of the expression data, including relative expression value and variance, of the 8799 genes in AMs from each Normal, Dex, or Dex-Pc rat. The principle component analysis (PCA) software examined three components of genes in different samples for those with similar or different expression profiles. The first component, shown in the x-axis, includes genes with a high degree of variance. The second component, displayed in the y-axis, encompasses genes that had a median range of variance. The third component, represented by z-axis, contains those with a minor variance.

Since each GeneChip contained 8799 probe sets, a total of 105,588 expression data points were generated from the twelve arrays. Principle component analysis (PCA) was first performed to examine the correlations among the data produced from different arrays. The results of the first three principal components, which included the variance of 83.5% of the expression data points of each sample, are shown in Fig. 1. Each blue, red, or green dot represents the overall expression pattern of each AM sample from Normal, Dex, or Dex-Pc rats, respectively (Fig. 1). The PCA analysis showed that the samples within each rat group were closely clustered together, whereas the samples between rat groups were distinctly separated, indicating that the quality of the microarray data was excellent. The PCA results also indicated that the global expression patterns in AMs of the same rat group were similar, whereas those in AMs of different rat groups were different.

### Hierarchical clustering analysis of differentially expressed genes

**Figure 2:**
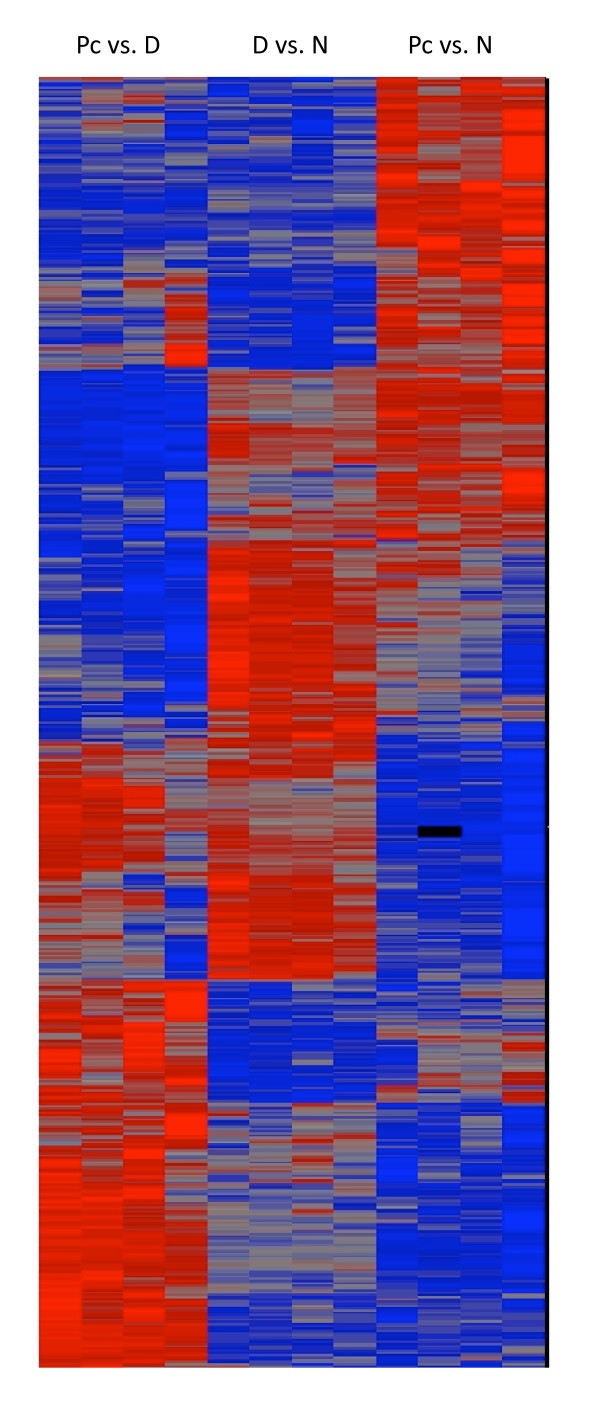
**Hierarchical clustering of differentially expressed genes**. ANOVA was first performed to identify genes that are differentially expressed due to dexamethasone treatment or *Pneumocystis *infection. Each lane represents the expression profile of AMs from one rat. The first four lanes show the expression profiles of AMs from the four Dex-Pc rats compared to that of Dex rats, the middle four lanes display those of the four Dex rats compared to that of Normal rats, and the remaining four lanes represent those of the four Dex-Pc rats compared to that of Normal rats. Red and blue colors indicate high and low expression levels, respectively. Gray color indicates no change in expression levels.

After ANOVA, 3473 genes were found to be differentially expressed due to dexamethasone treatment or *Pneumocystis *infection and were analyzed by hierarchical clustering using the Partek software (Fig. 2). Genes that were differentially expressed due to *Pneumocystis *infection were divided into four categories. The first one includes genes whose expressions were not affected by *Pneumocystis *infection. The second category includes those that were expressed at low levels but were up regulated by *Pneumocystis *infection. The third category contains genes that were expressed at high levels and were not affected by *Pneumocystis *infection. The fourth category includes those that were expressed at high levels but were down regulated by *Pneumocystis *infection. The same four categories of gene expressions in AMs from dexamethasone treated rats were observed.

### Functional pathways affected by dexamethasone or *Pneumocystis *infection

**Figure 3:**
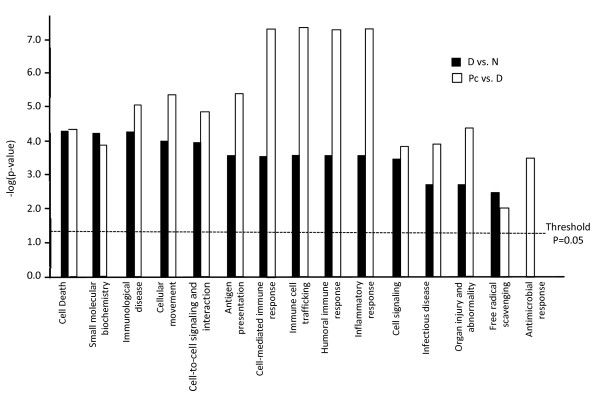
**Functions affected by dexamethasone or *Pneumocystis *infection**. Cellular functions identified by IPA as being affected by dexamethasone or *Pneumocystis *infection are illustrated with bar graphs based on the levels of -log(p-value), the higher the levels the more significant of the effect. Black bars indicate functions affected by dexamethasone treatment, while open bars denote those affected by *Pneumocystis *infection.

To ensure the accuracy of the results, only the differentially expressed genes with a false-discovery rate (FDR) ≤ 0.1 and a fold change (FC) ≥ 1.5 were further analyzed. With IPA, the following functions were found to be significantly affected by dexamethasone (listed in the order of significance from highest to lowest): cell death, small molecular biochemistry, immunological disease, cellular movement, cell-to-cell signaling and interaction, immune cell trafficking, antigen presentation, cell-mediated immune response, humoral immune response, inflammatory response, respiratory disease, cell signaling, infectious disease, organ injury and abnormality, and free radical scavenging. These functions were also affected by *Pneumocystis *infection, but in a different order of significance (also listed in the order of significance from highest to lowest): antigen presentation, cell-mediated immune response, humoral immune response, and inflammatory response were equally and most severely affected, followed by cellular movement, immune cell trafficking, immunological disease, cell-to-cell signaling and interaction, cell death, organ injury and abnormality, cell signaling, infectious disease, small molecular biochemistry, antimicrobial response, and free radical scavenging (Fig. 3).

**Figure 4:**
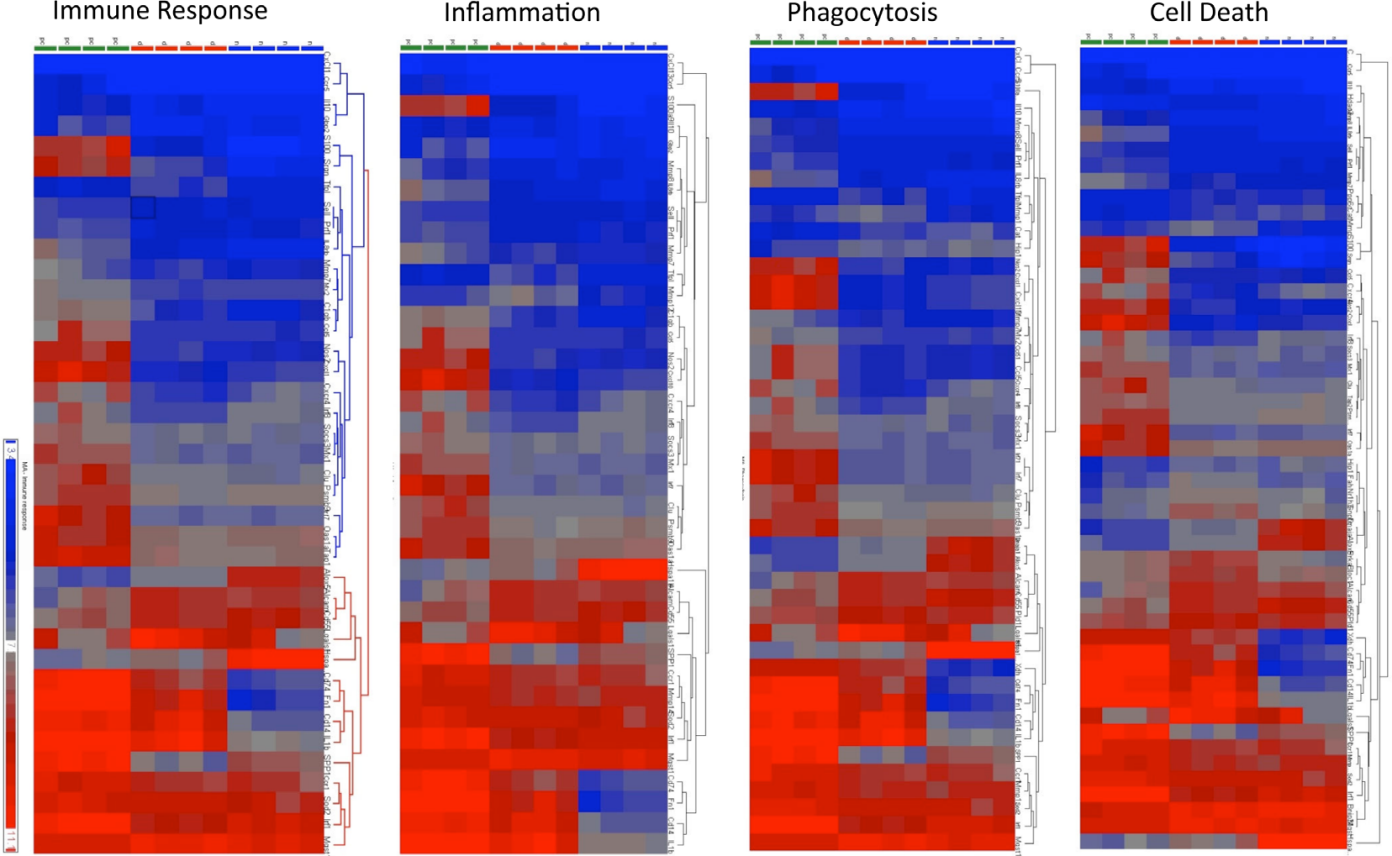
**Hierarchical clustering of differentially expressed genes related to the major functions of AMs**. Genes involved in immune response, inflammation, phagocytosis, and cell death were analyzed. Each lane represents the expression profile of AMs from one rat. For each panel, the first four lanes show the expression profiles of AMs from the four Dex-Pc rats compared to that of Dex rats, the middle four lanes display those of the four Dex rats compared to that of Normal rats, and the remaining four lanes represent those of the four Dex-Pc rats compared to that of Normal rats. Red and blue colors indicate high and low expression levels, respectively. Gray color indicates no change in expression levels.

The functions that were affected by *Pneumocystis *infection were further classified into four major groups: immune response, inflammation, cell death, and phagocytosis (Fig. 4). The immune response group included cell-mediated immune response, humoral immune response, and antigen presentation. The cell death group included cell death and organ injury and abnormality; while cell signaling, cell-to-cell interaction, cell movement, anti-microbial response, immune cell trafficking, and free radical scavenging were included in the phagocytosis group. Genes that were differentially expressed due to *Pneumocystis *infection not dexamethasone treatment in each group are shown in Table 1. It is interesting to note that these four functions share many of the same genes. Among these, Lgals1, Alcam, and Cd55 genes were down regulated; while Sod2, Soc3, Prf1, Il10, Mmp7, Sell, Psmb9, Oas1a, Clu, Ccr1, Mx1, Il8rb, Ccr5, Ccl5, Irf7, Nos2, and Cxcl10 genes were up regulated in all four functional groups. Cat and Hip1 genes that belong to both the cell death and phagocytosis groups were down regulated. In the cell death group, Hdac2, Bnip3L, Nr1h3, and Ppp6C genes were down regulated, and the Tap2 gene was up regulated. Mmp14 and Mmp8 in all but the immune response group, Mx2 in both immune response and phagocytosis groups, Gbp2 in both immune response and inflammation groups, and Tap1 in the immune response group were up-regulated.

**Table 1:** Genes involved in the four major AM functions affected by *Pneumocystis *infection

Gene	Pc vs. D	Immune Response (23 genes)	Inflammation (23 genes)	Cell Death (29 genes)	Phagocytosis (25 genes)
Lgals1	-4.24	↓	↓	↓	↓
Alcam	-2.29	↓	↓	↓	↓
Cd55	-1.68	↓	↓	↓	↓
Cat	-1.64	NA	NA	↓	↓
Hip1	-1.63	NA	NA	↓	↓
Hdac2	-1.61	NA	NA	↓	NA
Bnip3l	-1.58	NA	NA	↓	NA
Nr1h3	-1.52	NA	NA	↓	NA
Ppp6c	-1.52	NA	NA	↓	NA
Sod2	1.50	↑	↑	↑	↑
Socs3	1.67	↑	↑	↑	↑
Tap2	1.67	NA	NA	↑	NA
Mmp14	1.78	NA	↑	↑	↑
Prf1	1.78	↑	↑	↑	↑
Il10	1.87	↑	↑	↑	↑
Mmp7	1.92	↑	↑	↑	↑
Mx2	1.94	↑	NA	NA	↑
Sell	1.97	↑	↑	↑	↑
Psmb9	2.14	↑	↑	↑	↑
Oas1a	2.32	↑	↑	↑	↑
Mmp8	2.34	NA	↑	↑	↑
Clu	2.37	↑	↑	↑	↑
Ccr1	2.40	↑	↑	↑	↑
Mx1	2.42	↑	↑	↑	↑
Il8rb	2.78	↑	↑	↑	↑
Ccr5	2.79	↑	↑	↑	↑
Gbp2	3.21	↑	↑	NA	NA
Tap1	3.47	↑	NA	NA	NA
Ccl5	3.58	↑	↑	↑	↑
Irf7	4.92	↑	↑	↑	↑
Nos2	6.35	↑	↑	↑	↑
Cxcl10	12.33	↑	↑	↑	↑

Values shown are fold changes. Pc vs. D: expression affected by *Pneumocystis *(Pc) infection compared to the Dex (D) control. Up arrow (↑): up regulated by *Pneumocystis *infection; down arrow (↓): down regulated by *Pneumocystis *infection; NA: not applicable to the function.

Among the genes that were affected by dexamethasone and further affected by *Pneumocystis *infection, Mgst1 and Hspa1b genes were down-regulated, while Cd14, Irf8, Il1b, Cxcl13, Cxcr4, Fn1, Irf1, Cd74, S100a9, and Spp1 genes were up-regulated in all four groups (Table 2). The following genes were also up-regulated in some groups: Pld1 and Xdh in both cell death and phagocytosis; C1qb in both immune response and inflammation groups; Alox5 in all but the inflammation group; and Srgn in both immune response and cell death groups. Genes that were down-regulated in some groups include: Gnptg, Fah, Bloc1s2, and Prkacb in the cell death group; Dnaja1 in both cell death and phagocytosis groups; Tfp1 in all but the cell death group; Alox5 in all but the inflammation group; and Mmp12 in all but the immune response group.

**Table 2:** Genes involved in the four major AM functions affected by both dexamethasone and *Pneumocystis *infection

Gene	Pc vs. D	D. vs. N	Immune Response (16 genes)	Inflammation (15 genes)	Cell Death (22 genes)	Phagocytosis (18 genes)
Gnptg	-1.95	1.76	NA	NA	↓	NA
Fah	-1.80	1.50	NA	NA	↓	NA
Mmp12	-1.70	2.50	NA	↓	↓	↓
Dnaja1	-1.67	-3.20	NA	NA	↓	↓
Tfp1	-1.65	1.98	↓	↓	NA	↓
Bloc1s2	-1.63	1.61	NA	NA	↓	NA
Prkacb	-1.56	2.03	NA	NA	↓	NA
Alox5	-1.53	-3.07	↓	NA	↓	↓
Mgst1	-1.53	1.33	↓	↓	↓	↓
Hspa1b	-1.13	-13.90	↓	↓	↓	↓
Pld1	1.076	-1.05	NA	NA	↑	↑
Xdh	1.74	5.55	NA	NA	↑	↑
Cd14	1.85	8.10	↑	↑	↑	↑
Irf8	2.13	-1.61	↑	↑	↑	↑
Il1b	2.26	8.65	↑	↑	↑	↑
Cxcl13	2.41	4.17	↑	↑	↑	↑
C1qb	2.64	2.04	↑	↑	NA	NA
Cxcr4	3.60	-1.78	↑	↑	↑	↑
Fn1	4.20	10.19	↑	↑	↑	↑
Irf1	4.45	-1.52	↑	↑	↑	↑
Cd74	4.95	4.50	↑	↑	↑	↑
Srgn	5.34	3.39	↑	NA	↑	NA
S100a9	11.55	2.65	↑	↑	↑	↑
Spp1	11.78	-1.72	↑	↑	↑	↑

Values shown are fold changes. D vs. N: expression affected by dexathamethasone (D) treatment compared to the normal control (N); Pc vs. D: expression affected by *Pneumocystis *(Pc) infection compared to the Dex (D) control. Up arrow (↑): up regulated by *Pneumocystis *infection; down arrow (↓): down regulated by *Pneumocystis *infection; NA: not applicable to the function.

### Subcellular locations of differentially expressed genes

**Figure 5:**
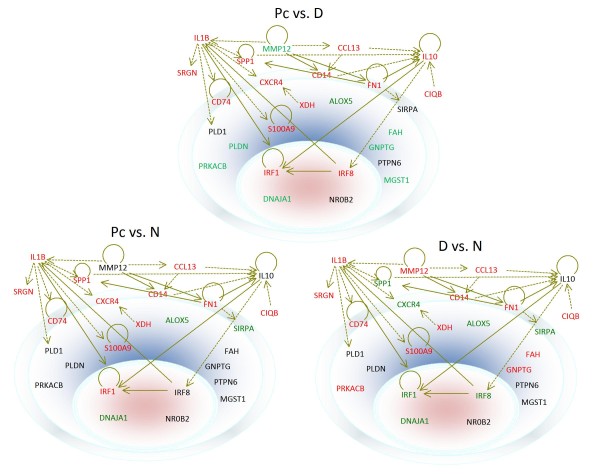
**Subcellular localization of the products of differentially expressed genes during dexamethasone treatment or *Pneumocystis *infection**. The outer ring represents the cell membrane, and the inner oval circle denotes the nucleus; the space between these two structures is the cytoplasm. Locations of the gene products are as indicated. Genes are shown in different colors, with red representing up-regulation and green down-regulation. Genes that have a direct relationship between each other are connected by solid arrows, and those with indirect relationships are linked by dotted arrows.

Among the proteins encoded by the genes whose expressions were affected by both dexamethasone and *Pneumocystis *in the four functional groups, IL1B, IL10, SRGN, MMP12, SPP1, and C1QB are secreted. CD74, CXCR4, SIRPA, FN1, and CD14 are membrane proteins, while MGST1, XDH, PLD1, S100A9, GNPTG, PTPN6, ALOX5, FAH, PLDN, and PRKACB proteins are located in the cytoplasm. IRF1, IRF8, DNAJA1, and NR0B2 are nuclear proteins (Fig. 5). Both IL-1B and IL-10 have a direct relationship with IRF1 and may affect its expression. IL-10 has an indirect relationship with IRF8, and IRF8 can regulate the expression of IL-1B. Except for Mgst1, Alox5, Fah, Pldn, Prkacb, Dnaja1, and Nrob2, all other genes are shown to have direct or indirect relationships between each other. This analysis also revealed four key proteins including IL-1B, IL-10, IRF1, and IRF8 that are central to the regulation of the differentially expressed genes in the four functional groups mentioned above.

### Effect of dexamethasone on AM gene expression (N vs. D)

When AM gene expression profiles between Normal and Dex (N. vs. D) groups were compared, 200 genes were found to be up-regulated and 144 genes were found to be down-regulated by dexamethasone treatment with an FDR ≤ 0.1 and FC ≥ 1.5 (Additional file 1, Tables S1 and S2). The top ten up-regulated genes were Cxcl2 (13.43), Fn1 (10.19), Ccl2 (9.99), Cd81 (9.07), Il1b (8.65), Trf (8.55), Slc28a2 (8.24), Cd14 (8.10), Cdh17 (7.15), and Sdc4 (6.52); and the top ten down-regulated ones were Hspa1a (-17.44), Hspa1b (-13.90), Hspb1 (-7.76), Hsph1 (-6.70), Tac1 (-6.16), Prkcb (-5.68), Atf3 (-4.91), Dnajb1 (-4.88), Fos (-4.54), and Ptprc (-3.92). Values in the parentheses are fold changes.

### Effect of *Pneumocystis *infection on alveolar macrophage gene expression (Pc vs. D)

Comparison of the expression profiles between Dex-Pc and Dex groups (Pc vs. D) revealed 116 genes up-regulated and 140 genes down-regulated by *Pneumocystis *infection (Additional file 1, Tables S3 and S4) also with the filter of FDR ≤ 0.1 and FC ≥ 1.5. The top ten up-regulated genes were Cxcl10 (12.33), Spp1 (11.78), S100A9 (11.55), Rsad2 (7.62), S100A8 (6.52), Nos2 (6.35), RT1-Bb (5.42), Lcn2 (5.36), RT1-Db1 (5.35), and Srgn (5.34); and the top ten down-regulated ones were Lgals1 (-4.24), Psat1 (-3.10), Tbc1d23 (-3.00), Gsta1 (-2.63), Car5b (-2.47), Xrcc5 (-2.35), Pdlim1 (-2.33), Alcam (-2.29), Cidea (-2.27), and Pkib (-2.25).

### Genes affected by dexamethasone but reversed by *Pneumocystis *infection

Since both dexamethasone and *P. carinii *infection have effects on gene expression in AMs, genes that were affected differently were examined. Thirty-two genes that were up regulated by dexamethasone treatment were reversely down regulated by *Pneumocystis *infection (Table 3). Another 32 genes that were up-regulated by dexamethasone were further up-regulated by *Pneumocystis *infection (Table 4). Nine genes that were down regulated by dexamethasone were found to be up regulated by *Pneumocystis *infection (Table 5), and twenty-one genes that were down-regulated by dexamethasone were further down-regulated by *Pneumocystis *infection (Table 6).

**Table 3:** Rat AM genes up-regulated by dexamethasone but down-regulated by *Pneumocystis *infection

Gene	D vs. N	Pc vs. D
Cdh17	7.15	-1.61
Gsta2	4.77	-2.63
Fxyd2	3.79	-1.97
Hsd11b1	3.19	-1.60
Diablo	2.72	-1.74
Mmp12	2.50	-1.70
Ccng1	2.36	-1.63
Btd	2.28	-1.85
Gaa	2.27	-1.60
Agt	2.25	-1.51
Hacl1	2.22	-2.13
Prkacb	2.03	-1.56
Pcsk1	2.01	-1.80
Tfpi	1.98	-1.65
Atp6v1d	1.96	-1.65
Hsd17b12	1.89	-1.61
Vldlr	1.82	-2.17
Hspa9	1.72	-1.72
Aco1	1.71	-1.85
Atp6v1a	1.69	-1.58
Tceb1	1.62	-1.62
Bloc1s2	1.61	-1.63
Tbc1d23	1.60	-3.00
Aifm1	1.57	-1.57
Gpd2	1.57	-1.54
Ufsp2	1.57	-1.51
Gnptg	1.56	-1.95
Sqstm1	1.56	-1.79
Hook1	1.55	-1.64
Plod1	1.52	-1.65
PVR	1.51	-1.68
Fah	1.50	-1.80

Values shown are fold changes. D vs. N: expression affected by dexathamethasone (D) treatment compared to the normal control (N); Pc vs. D: expression affected by *Pneumocystis *(Pc) infection compared to the Dex (D) control.

**Table 4:** Rat AM genes up-regulated by dexamethasone and further up-regulated by *Pneumocystis *infection

Gene	D vs. N	Pc vs. D
S100a9	2.65	11.55
S100a8	2.50	6.52
RT1-Bb	3.98	5.42
RT1-Db1	1.65	5.35
Srgn	3.39	5.34
Ass1	1.62	5.28
Apoe	2.47	5.23
Cd74	4.50	4.95
RT1-Da	6.15	4.35
Fn1	10.19	4.20
Il1r2	3.50	3.14
Enpp3	2.08	2.88
Slc28a2	8.24	2.71
F3	2.87	2.67
Ccl2	9.99	2.65
C1qb	2.04	2.64
Pon1	3.05	2.29
Il1b	8.65	2.26
Nudt4	3.48	2.15
Cd14	8.10	1.85
Ptafr	1.59	1.84
Arg1	1.60	1.83
Ptgs2	2.01	1.83
Pstpip1	3.29	1.79
Pde4b	1.88	1.76
Xdh	5.55	1.74
Socs2	1.73	1.67
Bst1	2.34	1.55
Gda	2.26	1.55
Ctsk	3.68	1.54
Emb	1.71	1.53
Ptpn1	2.46	1.50

Values shown are fold changes. D vs. N: expression affected by dexathamethasone (D) treatment compared to the normal control (N); Pc vs. D: expression affected by *Pneumocystis *(Pc) infection compared to the Dex (D) control.

**Table 5:** Rat AM genes down-regulated by dexamethasone but up-regulated by *Pneumocystis *infection

Gene	D vs. N	Pc vs. D
Spp1	-1.72	11.78
Irf1	-1.52	4.45
Cxcr4	-1.78	3.60
Crp	-1.86	3.23
Il1rn	-1.83	2.84
Irf8	-1.61	2.13
RT1-Aw2	-1.97	2.00
Ier3	-1.86	1.63
Ccnl1	-2.20	1.57

Values shown are fold changes. D vs. N: expression affected by dexathamethasone (D) treatment compared to the normal control (N); Pc vs. D: expression affected by *Pneumocystis *(Pc) infection compared to the Dex (D) control.

**Table 6:** Rat AM genes down-regulated by dexamethasone and further down-regulated by *Pneumocystis *infection

Gene	D vs. N	Pc vs. D
Alox5	-3.07	-3.07
Xrcc5	-1.92	-2.35
Hmgcs1	-1.78	-2.18
Gstm1	-1.72	-2.17
Hspa1a	-17.44	-2.08
Ela1	-1.62	-2.02
Ivns1abp	-1.88	-1.95
Igf1	-1.55	-1.81
Fbp1	-2.01	-1.77
Star	-1.85	-1.75
Dusp5	-2.40	-1.68
Dnaja1	-3.20	-1.67
Rgc32	-2.87	-1.67
Pparg	-1.56	-1.65
Dnajb1	-4.88	-1.59
Cd9	-1.54	-1.58
Ak3	-1.57	-1.57
St3gal2	-1.54	-1.56
Fcgrt	-2.15	-1.55
Mtpn	-1.62	-1.55
Cdc42ep3	-2.48	-1.52

Values shown are fold changes. D vs. N: expression affected by dexathamethasone (D) treatment compared to the normal control (N); Pc vs. D: expression affected by *Pneumocystis *(Pc) infection compared to the Dex (D) control.

### Confirmation of microarray results by RT-PCR

To ensure that the expression levels of genes determined by the microarrays were correct, real-time RT-PCR was performed on several selected target genes. Results confirmed that Cat was down-regulated and Cxcl10, Lcn2, Nos2, Sdc1, and Spp1 were up-regulated (Table 7). Genes whose expression levels were not significantly changed during PCP include Odc1, Smo, and RPS8.

**Table 7:** Confirmation of fold changes by real-time RT-PCR

Gene	**Microarray**^**a**^	**Real-time RT-PCR**^**b**^
Cat	-1.64	-3.50
Cxcl10	12.33	11.03
Lcn2	5.36	15.47
Nos2	6.35	14.58
Sdc1	2.42	16.50
Spp1	11.78	16.32

^a^Fold changes determined by microarray.

^b^Fold changes determined by real-time RT-PCR

## Discussion

In this study, DNA microarrays were used to study effects of *P. carinii *infection on global gene expression in AMs from rats. Since rats were immunosuppressed with dexamethasone in order to establish *Pneumocystis *infection, gene expression affected by dexamethasone treatment was also investigated. A total of 1682 genes in AMs were found to be affected by dexamethasone, and 1705 genes were found to be affected by *Pneumocystis *infection with an FDR of ≤ 0.1. With a more stringent filtering criteria of FDR ≤ 0.1 and FC ≥ 1.5, 200 genes were found to be up regulated and 144 genes down regulated by dexamethasone, and 115 genes were up regulated and 137 genes down regulated by *Pneumocystis *infection. Principle component analyses revealed that the results generated from the twelve microarrays were of excellent quality (Fig. 1). Because of costs, only one time point (eight weeks after organism inoculation) was examined in this study; this was a time when the Dex-Pc animals were heavily infected with the organism.

An *in vitro *microarray study had been conducted previously using the human A549 alveolar epithelial cells line [26]. The cells were incubated with *P. carinii *organisms for 2 hr and then analyzed for global gene expression with the Affymetrix human U95 Arrays. The results showed that some epithelial genes controlling cell cycle progression such as the ras-related *rho *gene and cyclin G-interacting protein gene were highly up-regulated by *P. carinii*. TNF-inducible protein and the *pim *oncogene that are involved in apoptosis signaling as well as inflammatory cytokines and chemokines including Gro-beta, IL-8, ICAM-1, MIP-3 and RANTES were also up-regulated [26]. Another microarray study was conducted by Hernandez-Novoa et al. [27]. They used total RNA from lung cells of wild type and CD40L knockout C57BL/6 mice infected with *P. murina *for various length of time (7 to 41 days) and found that 349 genes related to immune responses were up-regulated in wild type mice but not in CD40L-KO mice. The genes involved in innate response were up-regulated first followed by those involved in adaptive immunity. This study revealed how healthy, immunocompetent hosts respond to *Pneumocystis *infection [27]. In our study, we used AMs from *P. carinii*-infected rats to investigate how *Pneumocystis *affects AM functions by identifying genes that are up- or down-regulated during *Pneumocystis *infection.

IPA analyses showed that many cellular functions of AMs were affected by *Pneumocystis *infection (Fig. 3). Among them, antigen presentation, cell-mediated immune response, humoral immune response and inflammatory response were most profoundly affected. Up-regulation of genes involved in antigen presentation, such as Tap1, RT1-Bb and RT1-Db1, reflects the attempts AMs make to activate the adaptive immune responses. The observation that most genes involved in both cell-mediated and inflammatory responses were up regulated (Tables 1 and 2) implies that antigen presentation by AMs is functional during PCP. This postulation is consistent with that of Hernandez-Novoa et al. [27]. The fact that PCP progresses despite activation of cell-mediated immune response and inflammatory response indicates that other cellular functions are disabled. Due to the lack of appropriate antibodies, immunosuppression of rats is usually achieved by treatment with dexamethasone which is known to have an anti-inflammatory and a wide range of side effects. Results of this study revealed that in addition to the 1682 genes mentioned above, functions related to cell death, small molecule biochemistry (e.g., production of nitric oxide and reactive oxygen species), and immunological disease were most severely affected by dexamethasone (Fig. 3).

Dexamethasone and *Pneumocystis *infection were found to have opposite effects on certain genes. Of the 32 genes that were up-regulated by dexamethasone but down-regulated by *Pneumocystis *infection (Table 3), cadherin 17 (Cdh17) and glutathione-S-transferase alpha type2 (Gsta2) genes were most profoundly affected. Dexamethasone treatment increased Cdh17 expression by 7.15 fold, but *Pneumocystis *infection not only reversed this effect but also decreased its expression by 1.61 fold. Similarly, dexamethasone up-regulated Gsta2 by 4.77 fold, but *Pneumocystis *infection decreased it by 2.63 fold. Cadherin (calcium dependent adhesion molecule) plays a very important role in cell adhesion and assembly of the actin cytoskeleton [28]. Actin filaments are linked to α-catenin and to the cell membrane through vinculin which is linked to E-cadherin [29]. The decrease in cadherin expression during PCP may be a reason why AMs are defective in phagocytosis, as this function requires the actin cytoskeleton. Glutathione S-transferases (GSTs) link reduced glutathione via a sulfhydryl group to electrophilic centers on a variety of substrates [30]. This activity detoxifies compounds such as peroxidized lipids [31] that are generated during oxidative stress. The reduction in GST expression during PCP may explain the reduction in AM number as a decrease in GST expression would increase the concentration of toxic molecules such as reactive oxygen species [32] which can trigger apoptosis of AMs [33].

Equally important are genes that were down-regulated by dexamethasone but up-regulated by *Pneumocystis *infection. Among these genes (Spp1, Irf1, Cxcr4, Crp, Il1rn, Irf8, RT1-Aw2, Ier3, and Ccnl1) (Table 5), the secreted phosphoprotein 1 (Spp1) gene has the most dramatic reversal by *Pneumocystic *infection followed by interferon regulatory factor 1 (Irf1). The SPP1 protein is also known as bone sialoprotein, early T-lymphocyte activation (ETA-1), and most commonly osteopontin (Opn). Opn is one of the most abundantly expressed proteins in various lung diseases; it mediates diverse cellular functions such as adhesion, migration, and survival of several cell types including macrophages, T cells and dentritic cells [34,35]. OPN also functions as a Th1 cytokine, promotes cell-mediated immune responses, and plays a role in chronic inflammatory and autoimmune diseases and activation of immune cells [34]. Opn can be cleaved by thrombin to expose the sequence SVVYGLR which is a ligand of integrin receptors α4β1, α9β1, and α9β4 that are present on monocytes, macrophages, neutrophils, T cells, and mast cells [36,37]. Up-regulation of Opn during *Pneumocystis *infection may reflect the action of AMs in an attempt to activate both innate and adaptive immunities through the integrin receptors. Opn expression has been found to be up-regulated in AMs from smokers [38] and in titanium dioxide-induced lung disease in rats [39]; it is considered as a biomarker for particle-induced lung disease [39].

Both Irf-1 and Irf-8 genes were down-regulated by dexamethasone (-1.52 and -1.61, respectively) but up regulated by *Pneumocystis *infection (4.45 and 2.13 fold, respectively). IRF-1 is a transcription factor originally found to regulate the IFN-β gene family [40]. It also has other functions such as acting as a tumor suppressor [41], regulating the proliferation of smooth muscle cells, and activating the expression of iNOS [42]. In addition, IRF-1 induces transcription of genes such as PKR and 2',5'-oligoadenylate synthetase [43,44] that are involved in the defense against viral invasion. IRF-1 and IRF-8 are essential for proper functioning of mature macrophages. A defect in either gene results in the inability of the host to mount Th1-mediated immune response due to decreased production of IL-12 [45,46]. IRF-1 and IRF-8 together regulate numerous genes. Using microarrays, Dror et al. showed that 265 genes in activated macrophages are regulated by these two genes [47].

In addition to IRF1 and IRF8, IPA analyses revealed that IL-1 and IL-10 also play a major role in the regulation of gene expression during PCP. The expression of IL-1β was up-regulated 8.65 fold by dexamethasone and further up-regulated 2.26 fold by *Pneumocystis *infection (Table 4). IL-1 is a pro-inflammatory cytokine. Its up-regulation reflects the attempt of the host to combat the infection by inflammation. Two forms of IL-1 exist: IL-1α and IL-1β. IL-1 signals mainly through the type 1 IL-1 receptor (IL-1R1), leading to NF-κB and c-jun activation and expression of cytokines such as TNF-α and interferons, as well as other inflammation-related genes. IL-10 is an anti-inflammatory cytokine. It can repress the expression of inflammatory cytokines such as TNF-α, IL-6, and IL-1 by activated macrophages. The expression of IL-10 was not affected by dexamethasone treatment but was up-regulated 1.87 fold by *Pneumocystis *infection (Table 1). IL-10 has been shown to inhibit the expression of IL-1 receptor (IL-1R) gene [48] and up-regulate the expression of IL-1R antagonist [49]. Both actions would block the function of IL-1, thus decreasing production of pro-inflammatory cytokines. The fact that pro-inflammatory cytokines are produced despite IL-10 up-regulation suggests that the suppressive effects of IL-10 on IL-1 expression may be blocked during PCP.

Many (1705) genes are either up- or down-regulated in AMs during PCP. It is surprising that even though *Pneumocystis *is an extracellular pathogen, it is able to affect so many genes without getting into the cells. A number of cytokines such as TNF-α, IFN-γ, IL-1, IL-10, and IL-8 are over produced in the lung during PCP. They may affect the expression of these genes. *Pneumocystis *components such as the major surface glycoprotein or β-glucans that are present in the cell wall of the organism in large quantities may have some effects. Since *Pneumocystis *infection results in lung damage, cellular components released may also cause differential gene expression.

Among the top 10 up-regulated genes during PCP, the chemokine (C-X-C motif) ligand 10 (Cxcl10) gene was the most highly up-regulated one with a 12-fold increase in expression. CXCL10 binds to the chemokine receptor CXCR3 [50] and chemoattracts monocytes, macrophages, T cells, NK cells, and dendritic cells. It also promotes adhesion of T cells to endothelial cells [51,52]. The high degree of CXCL10 up-regulation suggests the attempts of the host to enhance AM phagocytosis. The other top up-regulated genes include Spp1, S100A9, Rsad2, S100A8, Nos2, RT1-Bb, Lcn2, RT1-Db1, and Srgn. These genes encode the secreted phosphoprotein 1 (SPP1), calgranulin A and B complex (S100A8/S100A9), radical S-adenosyl methionine domain containing 2 (RSAD2), inducible nitric oxide synthase (NOS2), class II MHC Bβ, lipocalin-2 (LCN2), class II MHC Dβ, and serglycin (SRGN) proteins, respectively.

As described above, the SPP1 protein plays a role in the activation of both innate and adaptive immunity. The calgranulin A and B complex (S100A8/S100A9) have been shown to be a damage-associated pattern molecule which mediates inflammatory responses and recruits inflammatory cells to sites of tissue damage [53]. It can also modulate polymerization of microtubules during migration of phagocytes and induces inflammatory responses in leucocytes and endothelial cells [54,55]. Their up-regulation in expression during PCP also shows the importance of phagocytosis in the defense against *Pneumocystis *infection. The RSAD2 protein is also known as viperin. It is an endoplasmic reticulum-associated, interferon-inducible virus inhibitory protein and has been shown to be required for optimal Th2 responses and T-cell receptor-mediated activation of NF-κB and AP-1 [56]. The NOS2 (iNOS) protein is responsible for the production of nitric oxide which is an antimicrobial compound [57].

The lipocalin-2 protein (LCN2) is a component of granules in neutrophils from tissues that are normally exposed to microorganisms. Its level is increased during inflammation [58]. LCN2 exerts bacteriostatic effects by its ability to capture and deplete siderophores that are small iron-binding molecules synthesized by certain bacteria as a means of iron acquisition [58]. Although *Pneumocystis *siderophores have not been identified and the role of LCN2 in PCP is unknown, iron is known to be essential for the proliferation of *Pneumocystis *[59], and deferoxamine, which is an iron chelator, has been used to treat PCP in animal models [59].

Serglycin (SRGN) is a proteoglycan mainly produced by hematopoietic and endothelial cells [60]. It plays an important role in the formation of several types of storage granules, especially in mast cells [61]. Serglycin can bind to a variety of molecules such as histamine, chymase, tryptase, and carboxypeptidase in mast cells; elastase in neutrophile; granzyme B in cytotoxic T cells; plasminogen activator in endothelial cells; and TNF-α in macrophages [60]. Serglycin is important for the retention of key inflammatory mediators inside storage granules and secretory vesicles [60]. Therefore, serglycin plays a role in inflammation which is also a host defense mechanism. RT1-Bb and RT1-Db1 are class II MHC molecules [62] and are involved in antigen presentation as described above. Their up-regulation also suggests the attempts of AMs to activate adaptive immunity.

Among the ten most down-regulated genes, the expression of the lectin, galactoside-binding, soluble, 1 (Lgals1) gene is most severely reduced by *Pneumocystis *infection. Lgals1 encodes galectin-1 which is an endogenous lectin that can trigger lymphocyte apoptosis [63]. Its down-regulation reflects the attempts of AMs to survive. The phosphoserine aminotransferase (Psat1) gene was the second most down-regulated gene. PSAT1 is over-expressed in colon tumors [64], but its role in PCP cannot be speculated due to limited information on its function. TBC1D3 is a member of the TBC1 domain family of proteins that stimulates the intrinsic GTPase activity of RAB5A, an essential actor in early endosome trafficking [65]. Its down-regulation would affect the phagocytic function of AMs. CAR5B is the mitochondrial form of carbonic anhydrase responsible for the inter-conversion of carbon dioxide and bicarbonate to maintain acid-base balance in blood and other tissues, and to help transport carbon dioxide out of tissues [66]. The active site of most carbonic anhydrases contains a zinc ion; therefore, they are classified as metalloenzymes. Although it was one of the most severely down-regulated genes, its role in PCP is not clear.

The X-ray repair complementing defective repair in Chinese hamster cells 5 (Xrcc5) gene encodes the Ku80 protein which is a helicase involved in DNA double-strand-break repair and chromatin remodeling [67]. Ku80 is also expressed on the surface of different types of cells and functions as an adhesion receptor for fibronectin [68] which enhances the interaction of AMs with *Pneumocystis *organisms [69]. Its down-regulation can be viewed as a double-edged sword as the inability of AMs to repair damaged DNA may trigger apoptosis thus decreasing their numbers, and the decrease in fibronectin receptor may decrease the phagocytic activity of AMs.

PDZ/LIM genes encode a group of proteins with diverse biological roles. In mammalian cells, there are ten genes that encode both a PDZ domain and one or several LIM domains [70]. All PDZ and LIM domain proteins can associate with and influence the actin cytoskeleton [71]. Down-regulation of any of these genes would affect the integrity of the actin cytoskeleton which plays a major role in phagocytosis. The activated leukocyte cell adhesion molecule (ALCAM) is an immunoglobulin superfamily cell adhesion molecule. It mediates both heterophilic (ALCAM-CD6) and homophilic (ALCAM-ALCAM) cell-cell interactions [72]. Its down-regulation in expression would affect the movement and thus phagocytic function of AMs.

The cell death-inducing DFF45-like effector (CIDE) family proteins include CIDEA, CIDEB, and CIDEC. These proteins are important regulators of energy homeostasis and are closely linked to the development of metabolic disorders including obesity, diabetes, and liver steatosis. CIDEA may initiate apoptosis by disrupting a complex consisting of the 40-kDa caspase-3-activated nuclease (DFF40/CAD) and its 45-kDa inhibitor (DFF45/ICAD) [73]. Its down-regulation can be viewed as the attempt of AMs to fight for survival by decreasing CIDEA-mediated apoptosis.

## Conclusions

Our data provide the first comprehensive description of the response of AMs to *Pneumocystis *infection using microarray and revealed a wide variety of genes and cellular functions that are affected by dexamethasone or *Pneumocystis *infection. Dexamethasone will continue to be used for immunosuppression if the rat PCP model is to be used for study of *Pneumocystis *infection. Knowing what dexamethasone will do to the cells will give investigators a better insight in studying the effect of *Pneumocystis *infection on gene expression and function of AMs. This study also revealed many defects of AMs that may occur during *Pneumocystis *infection, as many genes whose expressions are affected by the infection. Investigation of these genes will allow us to better understand the mechanisms of pathogenesis of PCP.

## Abbreviations

Aco1: aconitase 1; Agt: angiotensinogen; Aifm1: apoptosis-inducing factor, mitochondrion-associated 1; Ak3: adenylate kinase 3; Alcam: activated leukocyte cell adhesion molecule; Alox5: arachidonate 5-lipoxygenase; Apoe: apolipoprotein E; Arg1: arginase 1, liver; Ass1: argininosuccinate synthetase 1; Atf3: activating transcription factor 3; Atp6v1a: ATPase, H transporting, lysosomal V1 subunit A; Atp6v1d: ATPase, H+ transporting, lysosomal V1 subunit D; Azin1: antizyme inhibitor 1; Bloc1s2: biogenesis of lysosome-related organelles complex-1, subunit 2; Bnip3l: BCL2/adenovirus E1B interacting protein 3-like; Bst1: bone marrow stromal cell antigen 1; Btd: biotinidase; Calm1: calmodulin 1; C3: complement component 3; C1qb: complement component 1, q subcomponent, beta polypeptide; Car5b: carbonic anhydrase 5b, mitochondrial; Cat: atalase; Cxcl13: chemokine (C-X-C motif) ligand 13; Ccl2: chemokine (C-C motif) ligand 2; Ccl5: chemokine (C-C motif) ligand 5; Ccng1: cyclin G1; Ccnl1: cyclin L1; Ccr1: chemokine (C-C motif) receptor 1; Ccr5: chemokine (C-C motif) receptor 5; Cd14: CD14 molecule; Cd55: CD55 antigen; Cd74: CD74 antigen (invariant polypeptide of major histocompatibility complex, class I; Cd81: CD 81 antigen; Cd9: CD9 antigen; Cdc42ep3: CDC42 effector protein (Rho GTPase binding) 3; Cdh17: cadherin 17; Cidea: cell death-inducing DNA fragmentation factor, alpha subunit-like effector A; Clu: lusterin; Crp: C-reactive protein, pentraxin-related; Ctsk: cathepsin K; Cxcl1: chemokine (C-X-C motif) ligand 1; Cxcl10: chemokine (C-X-C motif) ligand 10; Cxcr4: chemokine (C-X-C motif) receptor 4; Diablo: diablo homolog; Dnaja1: DnaJ (Hsp40) homolog, subfamily A, member 1; Dnajb1: DnaJ (Hsp40) homolog, subfamily B, member 1; Dusp5: dual specificity phosphatase 5; Ela1: elastase 1; Emb: embigin; Enpp3: ectonucleotide pyrophosphatase/phosphodiesterase 3; F3: coagulation factor III; Fah: fumarylacetoacetate hydrolase; Fbp1: fructose-1,6- biphosphatase 1; Fcgrt: Fc receptor, IgG, alpha chain transporter; Fn1: fibronectin 1; Fos: FBJ osteosarcoma oncogene; Fxyd2: FXYD domain-containing ion transport regulator 2; Gaa: glucosidase, alpha, acid; Gbp2: guanylate nucleotide binding protein 2; Gda: guanine deaminase; Gnptg: N-acetylglucosamine-1-phosphotransferase, gamma subunit; Gpd2: glycerol-3-phosphate dehydrogenase 2, mitochondrial; Gsta2: glutathione-S-transferase, alpha type 2; Gstm1: glutathione S-transferase, mu 1; Hacl1: 2-hydroxyacyl-CoA lyase 1; Hdac2: histone deacetylase 2; Hip1: huntingtin interacting protein 1; Hmgcs1: 3-hydroxy-3-methylglutaryl-coenzyme A synthase 1; Hook1: hook homolog 1; Hsd11b1: hydroxysteroid 11-beta dehydrogenase 1; Hsd17b12: hydroxysteroid 17-beta dehydrogenase 12; Hspa1a: heat shock 70kD protein 1A; Hspa1b: heat shock 70 kD protein 1B; Hspa9: Heat shock protein 9; Hspb1: heat shock protein 1; Hsph1: heat shock 105 kDa/110 kDa protein 1; Ier3: immediate early response 3; Igf1: insulin-like growth factor 1; Il10: interleukin 10; Il1b: interleukin 1 beta; Il1r2: interleukin 1 receptor, type II; Il1rn: interleukin 1 receptor antagonist; IL8rb: interleukin 8 receptor, beta; Irf1: interferon regulatory factor 1; Irf7: interferon regulatory factor 7; Irf8: interferon regulatory factor 8; Ivns1abp: influenza virus NS1A binding protein; Lcn2: lipocalin 2; Lgals1: ectin, galactose binding, soluble 1; Mgst1: microsomal glutathione S-transferase 1; Mmp12: matrix metallopeptidase 12; Mmp14: matrix metallopeptidase 14; Mmp7: matrix metallopeptidase 7; Mmp8: matrix metallopeptidase 8; Mtpn: myotrophin; Mx1: myxovirus resistance 1; Mx2: myxovirus resistance 2; Nos2: nitric oxide synthase 2, inducible, macrophage; Nr1h3: nuclear receptor subfamily 1, group H, member 3; Nudt4: nucleoside diphosphate linked moiety X type motif 4; Oas1a: 2'-5' oligoadenylate synthetase 1A; Odc1: ornithine decarboxylase 1; Pcsk1: proprotein convertase subtilisin/kexin type 1; Pde4b: phosphodiesterase 4B, cAMP specific; Pdlim1: PDZ and LIM domain 1; Pkib: protein kinase inhibitor beta; Pld1: phospholipase D1; Plod1: procollagen-lysine, 2-oxoglutarate 5-dioxygenase 1; Pon1: paraoxonase 1; Pparg: peroxisome proliferator activated receptor gamma; Ppp6c: protein phosphatase 6, catalytic subunit; Prf1: perforin 1; Prkacb: protein kinase, cAMP dependent, catalytic, beta; Prkcb1: protein kinase C, beta; Psat1: phosphoserine aminotransferase 1; Psmb9: proteosome subunit, beta type 9; Pstpip1: proline-serine-threonine phosphatase-interacting protein 1; Ptafr: platelet-activating factor receptor; Ptgs2: prostaglandin-endoperoxide synthase 2; Ptpn1: protein tyrosine phosphatase, non-receptor type 1; Ptprc: protein tyrosine phosphatase, receptor type C; Pvr: poliovirus receptor; Rgc32: response gene to complement 32; Rps8: ribosomal protein subunit 8; Rsad2: radical S-adenosyl methionine domain containing 2; RT1-Aw2: RT1 class Ib, locus Aw2; RT1-Bb: class II histocompatibility antigen, B-1 beta chain; RT1-Da: RT1 class II, locus Da; RT1-Db1: RT1 class II, locus Db1; S100a8: S100 calcium binding protein A8 (calgranulin A); S100a9: S100 calcium binding protein A9 (calgranulin B); Sdc4: syndecan 4; Sell: selectin, lymphocyte; Slc28a2: solute carrier family 28, member 2; Smo: spermine oxidase; Socs2: suppressor of cytokine signaling 2; Socs3: suppressor of cytokine signaling 3; Sod2: superoxide dismutase 2, mitochondrial; Spp1: secreted phosphoprotein 1; Sqstm1: sequestosome 1; Srgn: serglycin; St3gal2: ST3 beta-galactoside alpha-2,3-sialyltransferase 2; Star: steroidogenic acute regulatory protein; Tac1: tachykinin 1; Tap1: transporter 1, ATP-binding cassette; Tap2: transporter 2, ATP-binding cassette; Tbc1d23: TBC1 domain family, member 23; Tceb1: transcription elongation factor B, polypeptide 1; Tfpi: tissue factor pathway inhibitor; Trf: transferrin; Ufsp2: UFM1-specific peptidase 2; Vldlr: very low density lipoprotein receptor; Xdh: xanthine dehydrogenase; Xrcc5: X-ray repair complementing defective repair in Chinese hamster cells 5.

## Authors' contributions

BHC, YL, and XX analyzed the microarray results. DL, CPL, MEL, and PJD performed the microarray experiments. CHL designed the experiments and wrote the manuscript. All authors read and approved the final manuscript.

## Supplementary Material

Additional file 1 **Table S1.** Rat alveolar macrophage genes up-regulated by dexamethasone. Table S2. Rat alveolar macrophage genes down-regulated by dexamethasone. Table S3. Rat alveolar macrophage genes up-regulated by *Pneumocystis *infection. Table S4. Rat alveolar macrophage genes down-regulated by *Pneumocystis *infection. Click here for file
