# Supplementary material for: Microarray studies on effects of Pneumocystis carinii infection on global gene expression in alveolar macrophages
Source: BMC Microbiol. 2010 Apr 8;10:103. doi: 10.1186/1471-2180-10-103 (PMC2858032; doi:10.1186/1471-2180-10-103)
Supplement: Additional file 1 — Table S1. Rat alveolar macrophage genes up-regulated by dexamethasone. Table S2. Rat alveolar macrophage genes down-regulated by dexamethasone. Table S3. Rat alveolar macrophage genes up-regulated by Pneumocystis infection. Table S4. Rat alveolar macrophage genes down-regulated by Pneumocystis infection. [file 1471-2180-10-103-S1.PDF]

Table S1. Rat alveolar macrophage genes up-regulated by dexamethasone

	**Gene**	**FC**	**FDR**
1.	Cxcl1	13.43	1.40E-05
2.	Fn1	10.19	1.68E-04
3.	Ccl2	9.99	9.25E-05
4.	Cd81	9.07	4.33E-06
5.	Il1b	8.65	5.35E-05
6.	Trf	8.55	2.84E-04
7.	Slc28a2	8.24	2.95E-05
8.	Cd14	8.10	2.39E-04
9.	Cdh17	7.15	1.41E-04
10.	Sdc4	6.52	5.06E-06
11.	RT1-Da	6.15	2.61E-05
12.	Tnf	6.14	1.33E-04
13.	Fcgr2b	6.09	2.22E-04
14.	Xdh	5.55	1.26E-05
15.	Crispld2	5.28	5.45E-04
16.	Cdo1	4.81	7.02E-04
17.	Gsta2	4.77	7.24E-06
18.	Gsta3	4.64	7.83E-06
19.	Cd74	4.50	2.26E-04
20.	RT1-Bb	3.98	3.26E-02
21.	Cxcl2	3.96	1.16E-04
22.	Ddah1	3.85	4.24E-04
23.	Fxyd2	3.79	7.53E-05
24.	Gusb	3.75	2.95E-05
25.	Ctsk	3.68	6.50E-05
26.	Ctsl	3.65	1.72E-05
27.	Il1r2	3.50	6.32E-03
28.	Nudt4	3.48	2.34E-03
29.	Srgn	3.39	2.52E-03
30.	Il1a	3.38	5.22E-04
31.	Pstpip1	3.29	6.44E-05
32.	Spry1	3.29	1.07E-03
33.	Oma1	3.25	3.26E-04
34.	Srprb	3.20	2.95E-03
35.	Hsd11b1	3.19	1.08E-05
36.	Ap1b1	3.17	2.43E-05
37.	Fabp4	3.13	4.15E-04
38.	Ly6c	3.12	1.37E-03
39.	Pon1	3.05	2.82E-02
40.	Itgam	3.02	2.98E-02
41.	B4galnt1	2.93	1.25E-03
42.	Orm1	2.89	1.59E-03

43.	F3	2.87	2.39E-02
44.	Emp1	2.86	4.05E-03
45.	Thrsp	2.86	9.83E-03
46.	Yc2	2.84	8.55E-05
47.	Ceacam10	2.82	1.40E-05
48.	Mgl1	2.79	4.86E-03
49.	Diablo	2.72	5.35E-05
50.	Mca32	2.71	4.29E-04
51.	Cst3	2.69	1.70E-04
52.	Akr1c18	2.66	2.73E-03
53.	S100a9	2.65	1.66E-02
54.	Slc6a8	2.65	4.89E-04
55.	Slc41a2	2.61	1.58E-03
56.	Mmp12	2.50	1.48E-03
57.	S100a8	2.50	3.39E-02
58.	Apoe	2.47	2.84E-02
59.	Syngr1	2.47	1.33E-04
60.	Ptpn1	2.46	7.03E-04
61.	Ampd3	2.44	1.23E-03
62.	Pf4	2.41	1.65E-04
63.	Slc1a3	2.41	2.67E-03
64.	Slc12a7	2.39	4.91E-05
65.	Slc16a1	2.38	3.66E-04
66.	Bid	2.36	1.66E-04
67.	Ccng1	2.36	2.82E-03
68.	Bst1	2.34	2.15E-04
69.	Btd	2.28	2.27E-03
70.	Gaa	2.27	4.47E-05
71.	Gda	2.26	2.67E-03
72.	Agt	2.25	1.16E-03
73.	Arsb	2.24	2.76E-05
74.	Cd36	2.24	3.37E-05
75.	Hacl1	2.22	3.08E-03
76.	Ccl4	2.19	1.48E-02
77.	Gns	2.15	1.40E-05
78.	Hk1	2.11	5.19E-04
79.	Cfd	2.09	1.40E-03
80.	Scarb1	2.09	4.83E-05
81.	Enpp3	2.08	8.47E-04
82.	C1qb	2.04	1.70E-02
83.	Cited2	2.04	6.68E-03
84.	Fdps	2.04	4.08E-02
85.	Hspa9	2.04	3.80E-02
86.	Prkacb	2.03	4.45E-04

87.	H1f0	2.01	3.67E-03
88.	Pcsk1	2.01	4.09E-05
89.	Ptgs2	2.01	4.52E-02
90.	Ccl3	1.98	5.22E-04
91.	Cpd	1.98	9.06E-03
92.	Tfpi	1.98	2.98E-03
93.	Mdh1	1.97	1.36E-04
94.	Ndufc1	1.97	4.39E-03
95.	Acsl4	1.96	1.63E-04
96.	Atp6v1d	1.96	2.25E-03
97.	Pik3ip1	1.96	7.99E-04
98.	Rab15	1.96	2.99E-03
99.	Brp44l	1.95	2.71E-04
100.	Ghr	1.94	9.71E-03
101.	Fabp5	1.93	6.45E-04
102.	Tpi1	1.93	6.89E-04
103.	Pgk1	1.91	7.31E-03
104.	Hsd17b12	1.89	3.74E-03
105.	Nme1	1.89	5.59E-03
106.	Nme2	1.89	2.15E-03
107.	Pde4b	1.88	4.34E-03
108.	Tmem171	1.86	1.73E-03
109.	Igf2r	1.85	4.88E-04
110.	Pdha1	1.84	1.66E-04
111.	Sqle	1.83	2.38E-02
112.	Tspo	1.83	1.60E-03
113.	Vldlr	1.82	6.46E-04
114.	Rraga	1.80	1.74E-02
115.	Slc11a2	1.79	1.10E-03
116.	Tmem126b	1.79	5.22E-03
117.	Gnptg	1.76	7.85E-03
118.	Pdia6	1.76	5.82E-03
119.	Acp2	1.74	4.15E-04
120.	Socs2	1.73	2.60E-03
121.	Rab13	1.72	3.00E-04
122.	Aco1	1.71	6.14E-03
123.	Btg3	1.71	1.58E-03
124.	Emb	1.71	5.09E-03
125.	P4hb	1.70	6.63E-04
126.	Prdx6	1.70	4.13E-03
127.	Tcn2	1.70	7.43E-04
128.	Atp6v1a	1.69	8.71E-03
129.	Atrn	1.69	1.31E-02
130.	Prkaa2	1.69	3.38E-02

131.	Txnip	1.67	3.36E-03
132.	Ifngr1	1.65	3.63E-02
133.	Ril	1.65	1.66E-02
134.	RT1-Db1	1.65	3.88E-02
135.	Mtch2	1.64	1.20E-03
136.	Ndufb2	1.64	9.73E-03
137.	Ap2a2	1.63	1.37E-03
138.	S100a10	1.63	1.19E-03
139.	Ass1	1.62	4.24E-03
140.	Snx4	1.62	3.67E-03
141.	Tceb1	1.62	4.61E-02
142.	Tspan8	1.62	8.49E-02
143.	Bloc1s2	1.61	2.77E-03
144.	Ndufb11	1.61	5.56E-03
145.	Arg1	1.60	4.13E-03
146.	Atp5j	1.60	4.02E-03
147.	Maf	1.60	3.36E-02
148.	Ndufb9	1.60	4.41E-03
149.	Ndufc2	1.60	2.14E-02
150.	Plekho1	1.60	2.15E-04
151.	Ssr4	1.60	5.21E-03
152.	Sts	1.60	1.60E-03
153.	Tbc1d23	1.60	2.46E-02
154.	Eef2k	1.59	2.42E-02
155.	Pfkl	1.59	3.16E-03
156.	Ptafr	1.59	4.39E-02
157.	Hdhd2	1.58	1.49E-02
158.	Twf1	1.58	3.25E-02
159.	Acat2	1.57	1.97E-02
160.	Aifm1	1.57	1.66E-02
161.	Cyp27a1	1.57	7.03E-04
162.	Gpd2	1.57	4.60E-02
163.	Ufsp2	1.57	1.17E-02
164.	Arf6	1.56	1.71E-03
165.	Lamp1	1.56	1.76E-03
166.	Ndufb8	1.56	3.51E-03
167.	Sqstm1	1.56	1.40E-02
168.	Acsl1	1.55	2.12E-02
169.	Hook1	1.55	4.77E-02
170.	Cox7a2	1.54	2.01E-03
171.	Ndufs8	1.54	1.92E-03
172.	Pcp4	1.54	6.50E-02
173.	Sel1l	1.54	5.38E-03
174.	Brcc3	1.53	3.06E-02

175.	Ctsb	1.53	5.74E-05
176.	Dbi	1.53	2.34E-02
177.	Ezr	1.53	1.24E-02
178.	Fuca1	1.53	2.71E-04
179.	Jtb	1.53	4.86E-03
180.	Map3k8	1.53	2.92E-02
181.	Edn1	1.52	4.96E-02
182.	Mgmt	1.52	3.46E-03
183.	Plod1	1.52	4.85E-03
184.	Qdpr	1.52	1.81E-02
185.	Tra1	1.52	9.08E-02
186.	Atp5g1	1.51	2.72E-02
187.	Atp5o	1.51	1.96E-02
188.	Eno1	1.51	1.20E-03
189.	Polr2i	1.51	1.10E-03
190.	Psmb5	1.51	4.73E-03
191.	PVR	1.51	5.35E-02
192.	Scd2	1.51	4.36E-02
193.	Serpinb9	1.51	3.16E-03
194.	Sh2b2	1.51	1.74E-02
195.	Apba3	1.50	6.53E-03
196.	Fah	1.50	3.15E-02
197.	Pgcp	1.50	2.46E-03
198.	Ppil3	1.50	1.56E-02
199.	Pttg1ip	1.50	8.55E-05
200.	Rpp21	1.50	1.93E-02

Table S2. Rat alveolar macrophage genes down-regulated by dexamethasone

	Gene	Fc	FDR
1.	Hspa1a	-17.44	3.82E-05
2.	Hspa1b	-13.9	4.91E-05
3.	Hspb1	-7.76	6.32E-05
4.	Hsph1	-6.70	1.86E-04
5.	Tac1	-6.16	7.61E-06
6.	Prkcb1	-5.68	1.40E-05
7.	Atf3	-4.91	2.43E-05
8.	Dnajb1	-4.88	1.70E-04
9.	Fos	-4.54	1.40E-05
10.	Ptprc	-3.92	7.08E-03
11.	Tgm1	-3.87	3.04E-05
12.	Sirpa	-3.63	2.91E-03
13.	Jun	-3.33	6.46E-05
14.	Dnaja1	-3.20	1.13E-04
15.	Kcnn3	-3.10	8.55E-05
16.	Alox5	-3.07	4.14E-05
17.	Ptpn12	-2.88	1.03E-03
18.	Rgc32	-2.87	2.14E-04
19.	Evl	-2.76	1.64E-06
20.	Alox15	-2.74	2.43E-02
21.	Ccnl1	-2.63	1.40E-05
22.	Klk6	-2.62	2.55E-05
23.	Id3	-2.61	6.63E-04
24.	Hsp90ab1	-2.60	1.15E-03
25.	Tgfbr1	-2.49	1.35E-02
26.	Cdc42ep3	-2.48	6.38E-04
27.	Rara	-2.47	4.15E-04
28.	Lincr	-2.46	6.48E-05
29.	Gas5	-2.44	2.95E-03
30.	Dusp5	-2.40	3.72E-04
31.	Cnksr3	-2.36	9.52E-05
32.	Hsp90aa1	-2.36	3.31E-03
33.	Svil	-2.33	1.31E-03
34.	Gpd1	-2.32	1.00E-03
35.	Msn	-2.32	2.25E-02
36.	Ucp3	-2.32	6.91E-05
37.	Dnaja4	-2.29	1.35E-04
38.	Mat2a	-2.26	4.00E-03
39.	Sfrs10	-2.25	6.34E-04
40.	Ddit3	-2.24	1.99E-03
41.	Cebpb	-2.17	1.73E-04
42.	Cxxc5	-2.16	4.33E-06

43.	Fyn	-2.16	9.12E-04
44.	Fcgrt	-2.15	1.65E-04
45.	Gmpr	-2.13	8.55E-05
46.	Myc	-2.12	4.15E-04
47.	Btg2	-2.11	5.34E-03
48.	Arhgap25	-2.09	1.86E-04
49.	Dusp1	-2.09	3.63E-03
50.	Prkch	-2.08	8.23E-04
51.	Hmox1	-2.03	1.30E-02
52.	Cast	-2.02	3.29E-02
53.	Eif4a2	-2.01	4.45E-04
54.	Fbp1	-2.01	2.60E-03
55.	Adam17	-1.99	1.06E-03
56.	Crp	-1.97	6.86E-02
57.	Olr1	-1.97	4.80E-02
58.	RT1-Aw2	-1.97	5.72E-03
59.	Cfb	-1.96	1.19E-03
60.	Ccnd3	-1.95	1.08E-05
61.	Dusp6	-1.94	1.36E-02
62.	Slc27a2	-1.94	3.40E-03
63.	Pygl	-1.93	1.50E-04
64.	Xrcc5	-1.92	5.66E-04
65.	Casp3	-1.91	2.21E-02
66.	Eef2	-1.91	2.75E-02
67.	MGC112715	-1.91	6.94E-04
68.	Nap1l1	-1.90	1.04E-02
69.	Rgs1	-1.90	8.20E-03
70.	Ivns1abp	-1.88	1.08E-02
71.	Sfpq	-1.88	6.89E-04
72.	Hgf	-1.86	3.81E-03
73.	Ier3	-1.86	1.27E-02
74.	Lphn2	-1.86	6.46E-04
75.	Sfrs3	-1.86	5.19E-04
76.	Star	-1.85	2.86E-03
77.	Fgr	-1.84	2.51E-03
78.	Tchh	-1.84	2.38E-02
79.	Il1rn	-1.83	1.00E-02
80.	Id1	-1.82	1.34E-02
81.	Pim3	-1.81	6.52E-05
82.	Ephx1	-1.80	6.94E-04
83.	Pde3b	-1.80	5.22E-04
84.	Cxcr4	-1.78	9.83E-02
85.	Dock8	-1.78	4.15E-04
86.	Hmgcs1	-1.78	5.47E-02

87.	Ccnd1	-1.75	2.40E-04
88.	App	-1.73	1.70E-03
89.	Cish	-1.73	5.32E-04
90.	Arrb2	-1.72	7.68E-03
91.	Camk1	-1.72	1.82E-03
92.	Clip2	-1.72	2.73E-03
93.	Gstm1	-1.72	1.65E-03
94.	Sdc3	-1.72	2.98E-03
95.	Spp1	-1.72	3.19E-02
96.	Stx5a	-1.72	2.16E-02
97.	Jak1	-1.71	1.80E-02
98.	Bhlhb2	-1.69	5.23E-03
99.	Ptbp1	-1.68	1.14E-02
100.	Tgfbr2	-1.68	1.80E-02
101.	Zfp36	-1.68	1.24E-02
102.	Arhgef7	-1.67	6.48E-05
103.	Gnas	-1.67	7.79E-04
104.	Itgb3	-1.67	7.79E-04
105.	Cmtm6	-1.66	1.70E-05
106.	Gdi1	-1.66	3.54E-02
107.	Gadd45a	-1.64	3.43E-03
108.	Sfrs11	-1.64	1.04E-02
109.	Sort1	-1.64	4.13E-02
110.	Ela1	-1.62	6.89E-04
111.	Marcks	-1.62	4.94E-02
112.	Mdfic	-1.62	5.61E-03
113.	Mtpn	-1.62	2.77E-02
114.	Ptpra	-1.62	3.90E-03
115.	Irf8	-1.61	8.24E-02
116.	Mapk14	-1.61	9.27E-02
117.	Nxf1	-1.61	1.46E-03
118.	Tsc22d1	-1.61	2.82E-02
119.	Gna12	-1.59	3.78E-02
120.	Nr1d1	-1.59	1.53E-03
121.	Nr1d2	-1.59	4.90E-03
122.	Wdr23	-1.59	3.83E-03
123.	Ccnd2	-1.58	1.80E-02
124.	Ak3	-1.57	1.22E-02
125.	Rxra	-1.57	1.66E-02
126.	Hspd1	-1.56	3.73E-02
127.	Id2	-1.56	4.57E-03
128.	Pparg	-1.56	3.59E-03
129.	Arg2	-1.55	1.23E-03
130.	Chp	-1.55	1.50E-02

131.	Igf1	-1.55	3.31E-02
132.	Cd9	-1.54	1.04E-02
133.	Cfp	-1.54	9.72E-03
134.	Hnrnpa1	-1.54	2.54E-02
135.	Jak2	-1.54	3.00E-02
136.	Mxd4	-1.54	2.70E-03
137.	St3gal2	-1.54	5.59E-03
138.	Jup	-1.53	2.54E-03
139.	Irf1	-1.52	8.09E-03
140.	Ptpre	-1.52	1.89E-03
141.	Rcn3	-1.52	4.36E-03
142.	Pscd1	-1.51	2.66E-03
143.	Ncl	-1.50	2.63E-02
144.	Nup62	-1.50	6.34E-04

Table S3. Rat alveolar macrophage genes up-regulated by *Pneumocystis* infection

	**Gene**	**FC**	**FDR**
1.	Cxcl10	12.33	9.32E-05
2.	Spp1	11.78	4.39E-05
3.	S100a9	11.55	3.65E-04
4.	Rsad2	7.62	1.16E-03
5.	S100a8	6.52	2.70E-03
6.	Nos2	6.35	6.23E-05
7.	RT1-Bb	5.42	2.02E-02
8.	Lcn2	5.36	2.70E-03
9.	RT1-Db1	5.35	2.67E-04
10.	Srgn	5.34	1.23E-03
11.	Ass1	5.28	1.80E-05
12.	Apoe	5.23	3.55E-03
13.	Cd74	4.95	4.18E-04
14.	Irf7	4.92	2.02E-04
15.	Irf1	4.45	3.10E-05
16.	RT1-Da	4.35	3.36E-04
17.	Fn1	4.20	4.36E-03
18.	RT1-Ba	4.11	1.35E-02
19.	Apoa4	3.80	3.07E-02
20.	Ifitm3	3.79	5.50E-05
21.	Cxcr4	3.60	5.72E-03
22.	Ccl5	3.58	2.75E-03
23.	Tap1	3.47	6.62E-05
24.	Crp	3.41	8.40E-03
25.	RT1-S3	3.35	2.02E-04
26.	Gbp2	3.21	5.72E-03
27.	Il1r2	3.14	1.39E-02
28.	Enpp3	2.88	3.21E-04
29.	Il1rn	2.84	1.47E-03
30.	Ccr5	2.79	1.32E-02
31.	Il8rb	2.78	4.88E-03
32.	LO	2.78	1.04E-02
33.	Slc28a2	2.71	3.74E-03
34.	F3	2.67	3.60E-02
35.	C3	2.65	3.21E-04
36.	Ccl2	2.65	2.58E-03

37.	C1qb	2.64	2.62E-03
38.	Cd38	2.61	1.15E-03
39.	Mx1	2.42	8.53E-04
40.	Sdc1	2.42	2.02E-04
41.	Tacstd1	2.41	5.97E-03
42.	Ccr1	2.40	1.27E-02
43.	Clu	2.37	3.62E-03
44.	Mmp8	2.34	5.55E-03
45.	Hp	2.32	2.25E-03
46.	Oas1a	2.32	5.56E-03
47.	Pon1	2.29	8.34E-02
48.	Il1b	2.26	1.17E-02
49.	Sftpa1	2.17	2.55E-02
50.	Nudt4	2.15	2.86E-02
51.	Psmb9	2.14	3.71E-04
52.	Irf8	2.13	1.69E-02
53.	RT1-Aw2	2.00	5.38E-02
54.	Cd24	1.98	1.92E-02
55.	Sell	1.97	4.73E-03
56.	Mx2	1.94	7.04E-04
57.	Mmp7	1.92	1.80E-02
58.	Thy1	1.90	3.25E-02
59.	Car4	1.88	1.35E-02
60.	Il10	1.87	7.34E-03
61.	Cd14	1.85	5.06E-02
62.	Ptafr	1.84	1.83E-02
63.	Arg1	1.83	2.70E-03
64.	Ptgs2	1.83	7.68E-02
65.	Il4ra	1.80	2.67E-04
66.	Stat1	1.80	3.21E-04
67.	Alpl	1.79	1.97E-02
68.	Hivep2	1.79	3.21E-04
69.	Plek	1.79	6.54E-03
70.	Pstpip1	1.79	5.51E-03
71.	Mmp14	1.78	6.74E-03
72.	Prf1	1.78	1.25E-03
73.	Ifng	1.77	9.44E-03
74.	Pde4b	1.76	8.50E-02
75.	Gzmb	1.74	3.01E-03

76.	Rbm39	1.74	8.34E-02
77.	Vcan	1.74	3.01E-03
78.	Xdh	1.74	7.50E-03
79.	Krt8	1.72	3.08E-02
80.	RT1-CE1	1.70	5.90E-04
81.	Sparc	1.70	8.84E-03
82.	Slfn2	1.68	3.01E-03
83.	Serpine1	1.67	9.45E-02
84.	Socs3	1.67	1.32E-02
85.	Tap2	1.67	8.68E-04
86.	Tnfrsf1b	1.66	5.05E-03
87.	Bat2d	1.64	7.37E-02
88.	C1r	1.64	1.87E-02
89.	Ier3	1.63	3.83E-02
90.	Kras	1.63	1.69E-02
91.	Marcks	1.63	6.57E-02
92.	Pglyrp1	1.63	1.97E-02
93.	Gstm2	1.62	3.54E-02
94.	Cx3cl1	1.60	1.80E-02
95.	Hla-dma	1.60	1.44E-03
96.	Pdpn	1.60	1.35E-02
97.	P2ry6	1.58	1.04E-02
98.	Tpm1	1.58	6.94E-02
99.	Tpm3	1.58	5.04E-02
100.	Ccnl1	1.57	5.57E-03
101.	Itgb7	1.57	3.95E-02
102.	Ninj1	1.57	4.63E-03
103.	Rbpsuh	1.57	1.03E-02
104.	Bst1	1.55	1.10E-02
105.	Gda	1.55	4.74E-02
106.	Ctsk	1.54	2.46E-02
107.	Emb	1.53	1.96E-02
108.	Kbtbd10	1.53	1.06E-02
109.	Nfkbia	1.53	5.06E-03
110.	Timp1	1.53	3.11E-02
111.	Pigr	1.52	3.19E-02
112.	Sftpd	1.52	9.55E-03
113.	Ptpn1	1.50	3.77E-02
114.	Ripk3	1.50	5.52E-03

115.	Sod2	1.50	9.66E-02

Table S4. Rat alveolar macrophage genes down-regulated by *Pneumocystis* infection

	Gene	FC	FDR
1.	Lgals1	-4.24	8.44E-02
2.	Psat1	-3.10	3.82E-03
3.	Tbc1d23	-3.00	8.68E-04
4.	Gsta2	-2.63	2.02E-04
5.	Car5b	-2.47	5.01E-03
6.	Xrcc5	-2.35	3.21E-04
7.	Pdlim1	-2.33	1.47E-03
8.	Alcam	-2.29	1.35E-02
9.	Cidea	-2.27	6.74E-03
10.	Pkib	-2.25	8.79E-03
11.	Pfkp	-2.19	7.34E-03
12.	Hmgcs1	-2.18	3.61E-02
13.	Gstm1	-2.17	3.05E-04
14.	Vldlr	-2.17	3.71E-04
15.	Hacl1	-2.13	6.74E-03
16.	Cyb5	-2.12	4.96E-03
17.	Prodh2	-2.10	5.38E-03
18.	Hspa1a	-2.08	8.03E-02
19.	Lipa	-2.06	4.49E-04
20.	Ggh	-2.03	2.00E-03
21.	Ela1	-2.02	2.67E-04
22.	Pdhx	-1.98	5.97E-03
23.	Slc15a2	-1.98	2.31E-03
24.	Fxyd2	-1.97	5.63E-03
25.	Acp5	-1.96	2.62E-03
26.	Gnptg	-1.95	1.38E-03
27.	Ivns1abp	-1.95	1.17E-02
28.	Slc5a6	-1.90	1.05E-02
29.	Nnt	-1.87	1.62E-02
30.	Aco1	-1.85	5.72E-03
31.	Btd	-1.85	1.34E-02
32.	Ech1	-1.84	7.42E-04
33.	Podn	-1.83	2.24E-02
34.	Fxc1	-1.82	1.22E-02
35.	Arpc5l	-1.81	1.04E-02
36.	Igf1	-1.81	2.40E-02

37.	Man1a	-1.81	5.32E-02
38.	Fah	-1.80	9.14E-03
39.	Pcsk1	-1.80	2.67E-04
40.	Sqstm1	-1.79	6.48E-03
41.	Gng5	-1.78	7.54E-03
42.	Pld1	-1.78	2.24E-03
43.	Fbp1	-1.77	1.05E-02
44.	Star	-1.75	1.03E-02
45.	Diablo	-1.74	2.96E-03
46.	Hspe1	-1.73	3.85E-02
47.	Spint2	-1.73	4.91E-04
48.	Hspa9	-1.72	1.05E-02
49.	Acly	-1.71	1.27E-02
50.	Cdh17	-1.71	7.18E-02
51.	Mmp12	-1.70	2.59E-02
52.	Acat1	-1.69	9.48E-03
53.	Cd55	-1.68	1.82E-02
54.	Dusp5	-1.68	9.05E-03
55.	PVR	-1.68	2.62E-02
56.	Dnaja1	-1.67	1.24E-02
57.	Rgc32	-1.67	1.39E-02
58.	Sfxn3	-1.67	2.96E-03
59.	Vat1	-1.67	1.64E-02
60.	Impa1	-1.66	5.51E-03
61.	Atp6v1d	-1.65	1.35E-02
62.	Plod1	-1.65	4.32E-03
63.	Pparg	-1.65	4.24E-03
64.	Tfpi	-1.65	1.83E-02
65.	Cat	-1.64	2.26E-02
66.	Hook1	-1.64	3.33E-02
67.	Tnfrsf8	-1.64	5.90E-04
68.	Acox1	-1.63	2.34E-02
69.	Bloc1s2	-1.63	5.01E-03
70.	Ccng1	-1.63	4.15E-02
71.	Wdfy3	-1.63	8.67E-03
72.	Ddt	-1.62	3.50E-03
73.	Sfxn1	-1.62	4.58E-03
74.	Tceb1	-1.62	4.82E-02
75.	Hdac2	-1.61	3.22E-02

76.	Hsd17b12	-1.61	1.98E-02
77.	Gaa	-1.60	2.26E-03
78.	Hsd11b1	-1.60	2.71E-03
79.	Idh3g	-1.60	1.29E-02
80.	Slc22a18	-1.60	1.29E-03
81.	Dnajb1	-1.59	8.67E-02
82.	Lpl	-1.59	1.48E-02
83.	Atp6v1a	-1.58	1.97E-02
84.	Bnip3l	-1.58	5.68E-03
85.	Ccdc53	-1.58	1.05E-02
86.	Cd9	-1.58	6.59E-03
87.	Csde1	-1.58	2.67E-02
88.	Mrpl24	-1.58	2.97E-02
89.	Tnfrsf21	-1.58	7.04E-04
90.	Aifm1	-1.57	2.15E-02
91.	Ak3	-1.57	1.68E-02
92.	Bckdhb	-1.57	2.45E-02
93.	Map4k1	-1.57	3.39E-02
94.	Acadl	-1.56	1.68E-02
95.	Pmpcb	-1.56	1.02E-02
96.	Prkacb	-1.56	8.17E-03
97.	Prkch	-1.56	1.44E-02
98.	Psmd2	-1.56	1.32E-02
99.	St3gal2	-1.56	1.69E-02
100.	Dnajb9	-1.55	5.93E-03
101.	Fcgrt	-1.55	2.70E-03
102.	Il8ra	-1.55	1.53E-02
103.	MGC112899	-1.55	5.87E-03
104.	Mtpn	-1.55	4.32E-02
105.	Gpd2	-1.54	4.99E-02
106.	Usp12	-1.54	2.65E-02
107.	Alox5	-1.53	1.05E-02
108.	Atp6v1f	-1.53	3.74E-03
109.	Bax	-1.53	1.86E-02
110.	Cuta	-1.53	1.66E-02
111.	Gphn	-1.53	2.37E-02
112.	Mgst1	-1.53	8.62E-04
113.	Nat1	-1.53	6.90E-02
114.	Pde6d	-1.53	1.80E-02

115.	Pld2	-1.53	9.33E-03
116.	Polr2f	-1.53	2.86E-02
117.	Tmem9b	-1.53	1.18E-02
118.	Abhd14b	-1.52	5.52E-03
119.	Cdc42ep3	-1.52	3.20E-02
120.	Cdc5l	-1.52	3.76E-03
121.	Nr1h3	-1.52	7.30E-02
122.	Ppp6c	-1.52	4.28E-02
123.	Rab6a	-1.52	7.34E-03
124.	Trim3	-1.52	3.01E-03
125.	Agt	-1.51	3.38E-02
126.	Ap2b1	-1.51	1.97E-02
127.	Btf3l4	-1.51	3.65E-02
128.	Crip2	-1.51	1.29E-02
129.	Etfa	-1.51	1.50E-02
130.	Ufsp2	-1.51	2.20E-02
131.	Wfdc6a	-1.51	1.04E-02
132.	Aplp2	-1.50	1.44E-03
133.	Gsr	-1.50	6.61E-03
134.	Higd1a	-1.50	3.25E-02
135.	Hsd17b10	-1.50	9.11E-03
136.	Prps2	-1.50	1.39E-02
137.	Slc2a1	-1.50	3.20E-02
